# The Influence of Long COVID on the Cardiovascular System and Predictors of Long COVID in Females: Data from the Polish Long COVID Cardiovascular (PoLoCOV-CVD) Study

**DOI:** 10.3390/jcm13247829

**Published:** 2024-12-22

**Authors:** Agata Bielecka-Dabrowa, Joanna Kapusta, Agata Sakowicz, Maciej Banach, Piotr Jankowski, Michał Chudzik

**Affiliations:** 1 Department of Preventive Cardiology and Lipidology, Medical University of Lodz, 90-419 Lodz, Poland; 2 Department of Cardiology and Congenital Diseases of Adults, Polish Mother’s Memorial Hospital Research Institute (PMMHRI), 93-338 Lodz, Poland; 3 Department of Internal Diseases, Rehabilitation and Physical Medicine, Medical University of Lodz, 90-647 Lodz, Poland; 4 Department of Medical Biotechnology, Medical University of Lodz, 90-419 Lodz, Poland; 5 Ciccarone Center for the Prevention of Cardiovascular Disease, Johns Hopkins University School of Medicine, Baltimore, MD 21205, USA; 6 Department of Internal Medicine and Geriatric Cardiology, Medical Centre of Postgraduate Education, 81-813 Warsaw, Poland

**Keywords:** women’s health, SARS-CoV-2, Long COVID, coronavirus, COVID complications

## Abstract

**Background/Objectives:** Female sex is one of the Long COVID (LC) risk factors; however, the LC predictors in females have not been established. This study was conducted to assess the influence of LC on the cardiovascular system and to assess the age-independent predictors of LC in females. **Methods**: Patient information and the course of the disease with symptoms were collected in women at least 12 weeks after COVID-19 recovery. The study participants were followed for 12 months. ECG monitoring, 24 h ECG monitoring, 24 h blood pressure monitoring, echocardiography, and biochemical tests were performed. **Results**: We studied 1946 consecutive female patients (age 53.0 [43.0–63.0] vs. 52.5 [41.0–63.0], *p* = 0.25). A more frequent occurrence of LC was observed in females with a severe SARS-CoV-2 infection (*p* = 0.0001). Women with LC compared to the control group had higher body mass index (*p* = 0.001), lower level of HDL cholesterol (*p* = 0.015), higher level of TG (*p* < 0.001) and higher TG/HDL ratio (*p* < 0.001), more often myocardial damage (*p* < 0.001), and lower LVEF (*p* = 0.01). LC women had more often QRS fragmentation, longer QTcB, and one of the ECG abnormalities. In a multivariate analysis in younger females with BMI > 24.8 kg/m^2^, TG/HDL ratio > 1.89 and severe course of COVID-19 and in older females, TG/HDL ratio > 1.89, lower LVEF, and also severe course of infection were independent LC predictors. **Conclusions**: Independent predictors of LC occurrence in women, regardless of age, are severe course of COVID-19 and TG/HDL ratio > 1.89. The presence of comorbidities and lifestyle before COVID-19 had no impact on the occurrence of LC in females regardless of age.

## 1. Introduction

In 2019, the first cases of infection with the SARS-CoV-2 virus, which causes the COVID-19 disease, were recorded in the city of Wuhan. Most patients who have recovered from the COVID-19 disease regain their initial state of health. However, in some people, symptoms vary in terms of intensity and duration [1]. This phenomenon has been called the “Long COVID” syndrome, defined by the National Institute for Health and Care Excellence (NICE) as the persistence of symptoms for more than 4 weeks after the onset of the acute phase of COVID-19 disease [2]. The most commonly reported symptoms include fatigue, muscle weakness, shortness of breath, and joint or chest pain, as well as difficulty concentrating and memory disorders [3,4].

Factors that correlate with long-term COVID-19 include the number of symptoms in the acute phase of SARS-CoV-2 infection, the length of hospitalization, and the occurrence of comorbidities [5,6]. Older age is also a risk factor for persistent symptoms after recovery from COVID-19. It was observed that elderly patients more often reported fatigue, musculoskeletal pain, and impaired lung function, which was most often associated with a slower ability to regenerate the body in these people after suffering from the acute phase of the disease [3,6]. In our earlier study, we showed that in healthy people, risk factors independently associated with the occurrence of LC are severe COVID-19, body mass index, and joint pain (arthralgia) [7]. Other studies have shown that persistent symptoms of COVID-19 may be related to endothelial dysfunction caused by the action of the SARS-CoV-2 virus on ACE2 receptors [7,8,9]. Although in several reports the authors suggest the possibility of a protective effect of female hormones on the course of COVID-19 disease [10,11,12,13], some studies confirm the increased incidence of chronic fatigue in women [14]. Long COVID (LC) has been a great challenge for healthcare systems worldwide. Therefore, one of the main issues now is to select the potential risk factors of LC in order to prevent, early diagnose, and optimally treat LC. In this study, we aim to assess the predictors of Long COVID in women that are age-independent.

## 2. Material and Methods

This observational study included 1946 patients who were part of the STOP-COVID registry (ClinicalTrials.gov identifier—NCT05018052). Patients included in this study completed a 3–6 month observation period, in outpatient medical care, in the years 2020–2023. The basis for the diagnosis of Long COVID was symptoms that appeared in the acute phase of the disease and, after a period of 3 months from COVID-19, still persisted and/or worsened [15]. Symptoms persisting after infection with the SARS-CoV-2 virus were assessed during a follow-up visit. This study was reported to the Bioethics Committee of the District Medical Chamber in Lodz, and on 23 June 2021, consent was obtained under the number K.B.-0115/2021. The inclusion criteria for this study were a confirmed diagnosis of COVID-19 (in accordance with the current guidelines of the Polish Ministry of Health), resolution of acute clinical symptoms, at least 2 weeks since the last symptoms, and age over 18 years. The exclusion criterion for this study was the presence of comorbidities (except obesity). Within 3 months after recovering from COVID-19, the patient visited an outpatient clinic, the so-called “0” visit, during which a physical assessment and a detailed clinical interview were performed, including information on the course of SARS-CoV-2 infection, comorbidities, and post-COVID-19 symptoms. Patients qualified for the study also underwent biochemical tests (lipidogram), 24 h Holter ECG monitoring using MOBILE HOLTER—Medicalgorithmics Polska Sp. z o.o., 24 h ambulatory blood pressure monitoring (ABPM) using BTL—BTL Industries Limited, and 12-lead electrocardiogram (ECG) and echocardiography using the modified biplane Simpson’s rule to determine the ejection fraction and volume of the left ventricle, as well as tricuspid annular systolic excursion (TAPSE) to assess the right ventricle—in accordance with the guidelines of the European Society of Cardiology (ESC). In selected patients, an MR scan was performed using Intellispace Cardiac Tool (Philips NE). Cardiovascular magnetic resonance (CMR) scans were performed on two, three, or four 1.5 T MRI scanners. Images used a cardiographic vector technique for electrocardiogram (ECG) synchronization. The assessment protocol for biventricular function and volumes adhered to the published guidelines from the Society for Cardiovascular Magnetic Resonance (SCMR) regarding CMR protocols in COVID-19. The sequences implemented included steady-state free precession cine imaging, late gadolinium enhancement (LGE), first-pass perfusion, T1 (pre- and post-contrast) and T2 mapping, and T2-weighted short-tau triple inversion recovery.

Participants were also grouped based on the severity at the time of initial COVID-19 diagnosis using definitions of severe COVID-19 course [15] when they noticed one of the following:

- Hospitalization with diagnosis of pneumonia, respiratory failure, assisted breathing, or thromboembolic complications during hospitalization.

and/or

- Home course with symptoms lasting > 14 days, subjective evaluation by the patient as severe (“3” on a scale of 1–3), temperature > 38 °C, dyspnea, or saturation below 94 lasting more than 3 days.

Participants were grouped based on the severity at the time of initial COVID-19 diagnosis and also using definitions of “very light” COVID-19 course when they did not notice temperature > 38 °C, dyspnea, or saturation below 94 lasting more than 3 days, “light” when there was observed one of these symptoms, and “medium” if two of these symptoms were present.

The study participants were divided into two groups based on the occurrence of Long COVID. The LC group included 1227 patients with persistent symptoms after recovery from SARS-CoV-2, while the non-LC group included 719 patients without persistent symptoms. We also divided the women based on the median age (53 years old) and presence of LC to assess the predictors, regardless of age, of the LC occurrence.

### Statistical Analysis

The Polish version of Statistica v. 13 (Statsoft) was used to perform the statistical analysis. Results were presented as mean and standard deviation or median (25th–75th percentile). When analyzing the obtained data, the Shapiro–Wilk test (normal distribution), Student’s *t*-test (independent variables), and the Mann–Whitney U test or χ^2^ test with Yates’ correction were used, where appropriate. The significant parameters in the univariable analyses were introduced to the multivariable-logistic-regression models. Values of *p* < 0.05 were considered statistically significant.

## 3. Results

### 3.1. Evaluation of Basic Characteristics

The female patients were divided into two groups: 1227 in the LC group with median age 53 (43–63) and 719 patients with median age 52.5 (41–63) without LC. We also divided the women based on the median age (53 years old) and presence of LC—618 women with LC compared to 376 women without LC with median age < 53 and 609 women with LC compared to 343 women without LC with median age > 53—to assess the predictors, regardless of age, of the LC occurrence. The results of the analyses are presented in Tables S1–S10, which are part of the Supplementary Materials.

Women with LC compared to the control group had higher body mass index (27 vs. 26 kg/m^2^; *p* = 0.001), lower level of HDL cholesterol (59 vs. 60 mg/dL; *p* = 0.015), higher level of TG (100 vs. 89 mg/dL; *p* < 0.001), and higher TG/HDL ratio (1.72 vs. 1.50; *p* < 0.001). The clinical characteristics of the studied groups are presented in Table 1.

The presence of investigated comorbidities such as hypertension, diabetes, or coronary artery disease did not affect the occurrence of LC (Table 2).

### 3.2. Evaluation of the Course and Symptoms During and Post-COVID-19

In females with a severe course of COVID-19 in the acute phase of the disease, Long COVID was observed more often: 35% vs. 23% (*p* = 0.0001). Moreover, when analyzing the symptoms occurring during COVID-19 disease in the LC group, patients reported the following symptoms compared to controls: fatigue, memory and concentration disturbances, anosmia and ageusia, hair loss, dyspnea, musculoskeletal pain, headache, sleep disorders, neurosis, and depression (*p* < 0.001 for all variables)—Table 3.

### 3.3. The Assessment of the Lifestyle Influence on Long COVID

Lifestyle parameters before the onset of COVID-19, such as smoking, alcohol abuse, and physical activity had no impact on the occurrence of LC (Table 4).

### 3.4. The Comparison of the Echocardiographic and Magnetic-Resonance Parameters in Women with and Without Long COVID

Women with LC compared to the control group had more often myocardial damage assessed in late-gadolinium-enhancement cardiac magnetic resonance (CMR) (6.2 vs. 2.9%; *p* < 0.001). LC women had also lower LVEF (60 vs. 63%; *p* = 0.01)—Table 5.

### 3.5. The Differences in 24 h ECG and ABP Monitoring and ECG Examination Between Women with and Without Long COVID

LC women had more often QRS fragmentation, longer QTcB, higher minimal day HR, and one of the ECG abnormalities: HR >100/min, QRS > = 120 ms, ST-T changes, T inversion, arrhythmia, and QRS fragmentation in ECG examination. There were no significant differences regarding other 24 h ECG and 24 h ABPM parameters between assessed groups—Table 6.

### 3.6. Multivariate Regression Analysis

**Figure 1:**
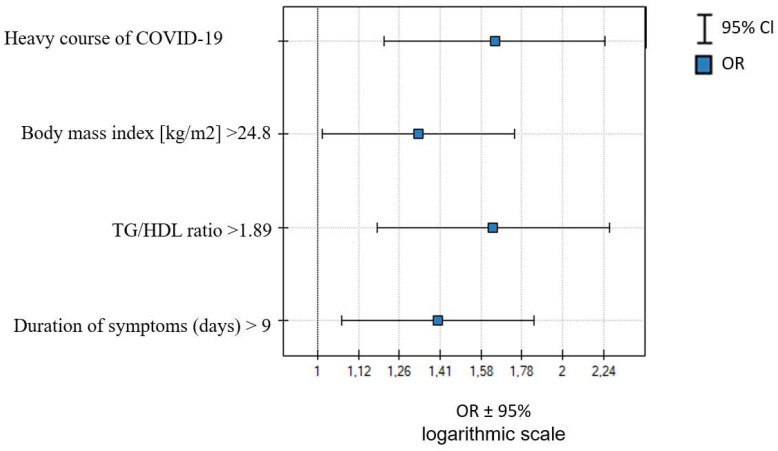
Independent predictors of LC occurrence in women with median age < 53 years old.

In a multivariate analysis in younger females (median age < 53 years), the logistic regression was conducted with the step-back analysis. BMI > 24.8 kg, Tg/HDL ratio > 1.89, severe course of COVID-19, and duration of infection >9 days were independent LC predictors—Table 7 and Figure 1.

**Figure 2:**
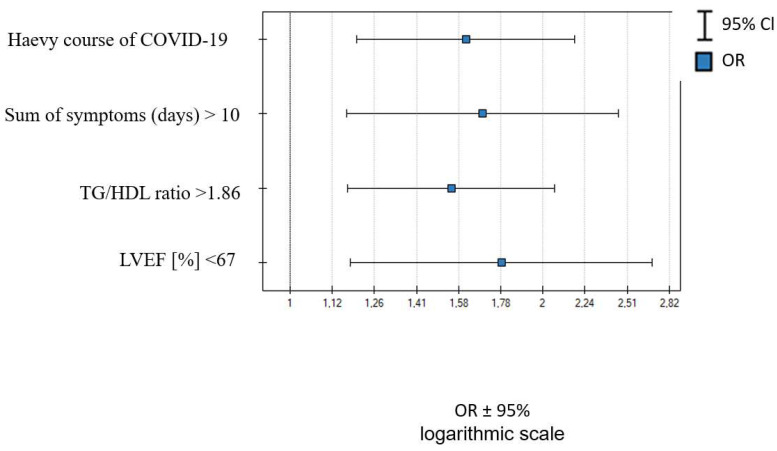
Independent predictors of LC occurrence in women with median age > 53.

In females > 53 years old, Tg/HDL ratio > 1.89, LVEF < 67%, severe course of COVID-19, and sum of symptoms during infection >10 were independent LC predictors—Table 8 and Figure 2.

## 4. Discussion

In our study, women with LC compared to the control group had higher body mass index, lower level of HDL cholesterol, and higher level of TG, as well as higher TG/HDL ratio. LC in women was also significantly correlated with a severe course of SARS-CoV-2 infection. Women with LC have more often myocardial damage assessed in late-gadolinium-enhancement CMR, lower LVEF, and ECG abnormalities. What is interesting is that the presence of investigated comorbidities, such as hypertension, diabetes, or coronary artery disease, did not affect the occurrence of LC, as well as lifestyle parameters before the onset of COVID-19, such as smoking, alcohol abuse, and physical activity, also had no impact on the occurrence of LC. Independent predictors of LC occurrence in women, regardless of age, were severe course of COVID-19 and Tg/HDL ratio > 1.89.

Recent studies on a large group of patients have confirmed that female sex increases the risk of post-COVID-19 syndrome [16,17]. Three other multicenter studies have also found it to increase the likelihood of developing some post-COVID-19 symptoms, such as fatigue, shortness of breath, and dermatological problems [18,19,20,21]. In a meta-analysis of observational studies by Gennaro, based on 120,970 participants (mean age: 52.3 years; 48.8% females) who were followed up for a median of six months, the incidence of any Long COVID symptomatology was 56.9% (95% CI 52.2–61.6). Higher mean age was associated with higher incidence of psychiatric, respiratory, general, digestive, and skin conditions [22]. Also, in a prospective cohort study by Bai et al., female sex was independently associated with Long COVID syndrome in a multivariable analysis, and advanced age and active smoking were also associated with a higher risk of Long COVID [23]. In another study with data of 1.2 million individuals (from 22 countries) who had symptomatic SARS-CoV-2 infection, the Long COVID symptom clusters were more common in women aged 20 years or older (10.6% (95% CI, 4.3–22.2%)) 3 months after symptomatic SARS-CoV-2 infection than in men aged 20 years or older (5.4% (95% CI, 2.2–11.7%)). Both sexes younger than 20 years of age were estimated to be affected in 2.8% (95% CI, 0.9–7.0%) of symptomatic SARS-CoV-2 infections [15,24]. In our study, 63% of females presented Long COVID. In the study of Loosen SH et al. based on 50,402 COVID-19 patients in the Disease Analyzer database (IQVIA) featuring data from 1056 general practices in Germany, lipid metabolism disorders (OR 1.46, 95% CI 1.28–1.65, *p* < 0.001) and obesity (OR 1.25, 95% CI 1.08–1.44, *p* = 0.003) were strong risk factors for the development of Long COVID [25]. Triglyceride (TG) to high-density lipoprotein (HDL) ratio values > 2.75 in men and > 1.65 in women were found in the Metabolic Syndrome in Active Subjects (MESYAS) study—18,778 active workers enrolled in three insurance companies in Spain—to be highly predictive of the metabolic syndrome (MS) diagnosis. TG/HDL ratio was also found to have a high predictive value of a first coronary event regardless of body mass index (BMI) [26]. In our study, Tg/HDL ratio > 1.89 was the independent predictor of Long COVID occurrence in women regardless of age. Regardless of age, lipid metabolism abnormalities increase the risk of persistent symptoms after COVID-19 in females, suggesting that metabolic alterations determine the risk for unfavorable disease courses along all phases of COVID-19.

In the diagnosis of patients after SARS-CoV-2 infection, abnormalities in the electrocardiographic (ECG) examination can provide a lot of important information. It has been observed that the severity of the course of COVID-19 was influenced by abnormalities recorded in the ECG during the patient’s admission to the hospital, such as QRS fragmentation (fQRS) in the 12-lead ECG, T-wave inversion, or ST segment depression [27]. Furthermore, the analysis conducted by De Vita et al. showed that QRS duration, ST segment depression, and the presence of any abnormalities in the electrocardiographic examination on admission were independently associated with increased mortality in patients with COVID-19 [28]. Siedlecki et al. presented the most common ECG changes in the course of COVID-19, which included pathological changes in atrioventricular conduction, P wave, and QRS abnormalities [29]. In our analysis, QRS fragmentation, longer QTcB, and one of the ECG abnormalities were more frequently observed in women with Long COVID. Imaging studies (CMR) confirmed more frequent myocardial damage in women with LC compared to the control group (6.2 vs. 2.9%; *p* < 0.001) and lower LVEF (60 vs. 63%; *p* = 0.01).

In our previous study [30] conducted in a group of 886 patients (443 women and 443 men), we observed that women, regardless of age and body mass index, more often than men complained about persistent symptoms after COVID-19 a year after recovery from COVID-19. Moreover, women after COVID-19 had lower mean arterial pressure and pulse pressure in 24 h ABPM, fewer ECG abnormalities, and more often higher heart rate and arrhythmia in 24 h ECG monitoring than men. Wang K. et al. and Moscucci F. et al. observed that female patients have increased expression and activation of angiotensin type 2 receptors, which participate in the immune response against SARS-CoV-2 infection and are involved in the regulation of blood pressure and kidney function, providing protection against cardiovascular complications [31,32]. In premenopausal women, higher levels of ACE2 and increased activity of the sex-hormone-dependent ACE2 pathway may contribute to a reduced incidence of cardiovascular disease [33,34]. In the postmenopausal period, however, due to reduced levels of sex hormones (progesterone and estrogen), women are more vulnerable to severe COVID-19 outcomes and may also experience long-term COVID-19 symptoms. This is associated with impaired immune regulation and increased susceptibility to infections [35]. In a cross-sectional study (Mount Sinai–Yale Long COVID; MY-LC) conducted in a group of 275 subjects with Long COVID, as many as 268 people had significantly lower cortisol levels [36]. Hormonal imbalance in women may be associated with a longer recovery. Because Long COVID is a new disease entity, the impact of which has not yet been fully established, it is therefore necessary to collect as much research data as possible and develop targeted treatment strategies [37,38].

### Strength and Limitations of the Study

So far, the need for screening for cardiovascular complications in patients after COVID-19 has not been clearly demonstrated. Therefore, until appropriate guidelines are developed, the diagnostic procedure should be carried out in an individualized manner, taking into account the course of the acute phase of COVID-19 and clinical symptoms reported or observed after infection. We are aware of the limitations of our study, such as the relatively short observation period. Extending the observation period could provide equally interesting information regarding the persistence and impact of Long COVID on the cardiovascular system and the identification of age-independent predictors of Long COVID. Therefore, it is necessary to collect as much research data as possible. The STOP-COVID program is still ongoing, and further analyses are planned in the future. In addition, our study was conducted in a public medical facility, where most patients were treated on an outpatient basis due to COVID-19. Unfortunately, the facility did not have the option of including spirometry or diffusion capacity testing; instead, patients compared their perception with previous respiratory function from a subjective perspective. To minimize the risk of potential factors influencing the diagnosis, such as comorbidities or symptoms present before SARS-CoV-2 infection, the diagnosis placed very carefully. In the case of clinical suspicion of disorders and/or abnormal results in resting and exercise pulse oximetry, patients were referred for further pulmonary diagnostics.

## 5. Conclusions

Independent predictors of LC occurrence in women, regardless of age, are severe course of COVID-19 and Tg/HDL ratio > 1.89. The presence of comorbidities and lifestyle before COVID-19 had no impact on the occurrence of Long COVID in females regardless of age.

## Figures and Tables

**Table 1:** The clinical characteristics—differences between females with and without Long COVID.

Variable	Women with Long COVID (n = 1227)	Women Without Long COVID (n = 719)	*p*
Clinical characteristics			
Age	53.00 (43.00–63.00)	52.50 (41.00–63.00)	0.25
Weight (kg)	72.00 (64.00–83.00)	70.00 (62.00–80.00)	0.001
Height (cm)	164.00 (160.00–168.00)	164.00 (160.00–168.00)	0.95
Body mass index (kg/m^2^)	26.77 (23.72–30.80)	26.03 (22.83–29.75)	0.001
No comorbidities	806 65.7%	463 64.4%	0.56
Symptomatic course of COVID-19	1157 94.3%	688 95.7%	0.18
Home isolation	1019 83.0%	863 82.8%	0.55
Hospitalization with pneumonia	117 9.5%	49 6.8%	0.03
Biochemical parameters			
TC (mg/dL)	196.00 (168.50–224.00)	195.00 (170.00–221.00)	0.67
HDL (mg/dL)	59.00 (50.00–66.00)	60.00 (51.00–68.00)	0.01
LDL (mg/dL)	116.00 (90.00–141.00)	115.00 (92.00–137.00)	0.49
TG (mg/dL)	100.00 (74.00–139.00)	89.00 (68.00–125.30)	0.001
Non-HDL (mg/dL)	136.00 (110.00–163.00)	134.00 (109.00–158.00)	0.24
TG/HDL	1.72 (1.16–2.60)	1.49 (1.00–2.35)	<0.001
Glucose (mg/dL)	96.00 (90.00–104.00)	96.00 (89.00–103.00)	0.46
D-dimers (µg/L)	0.36 (0.24–0.52)	0.36 (0.23–0.52)	0.91
Vitamin D_3_ level (µg)	29.15 (22.00–39.00)	29.00 (22.00–40.63)	0.80
Calcium (mg/dL)	9.46 (9.16–9.70)	9.36 (9.11–9.61)	0.11

Abbreviations: TC—total cholesterol, HDL—high-density lipoprotein cholesterol, LDL—low-density lipoprotein cholesterol, TG—triglyceride.

**Table 2:** Comorbidities in females with and without Long COVID.

Variable	Women with Long COVID (n = 1227)	Women Without Long COVID (n = 719)	*p*
Clinical characteristics			
HA	418 34.1%	240 33.4%	0.75
DM type 2	119 9.7%	58 8.1%	0.22
Coronary artery disease	63 5.1%	28 3.7%	0.20
Myocardial infection in the past	18 1.5%	8 1.1%	0.51
Cardiomyopathy	4 0.33%	2 0.27%	0.85
Hyperlipidaemia	240 19.6%	123 17.1%	0.18
Asthma	133 10.8%	69 9.6%	0.38
COPD	22 1.8%	12 1.7%	0.84
Thyroid disease	254 20.7%	162 22.5%	0.34
Hashimoto disease	190 15.5%	100 13.9%	0.34

Abbreviations: HA—arterial hypertension, DM—diabetes mellitus, COPD—chronic obstructive pulmonary disease.

**Table 3:** The course and symptoms during COVID-19 in females with and without Long COVID.

Variable		Women with Long COVID (n = 1227)	Women Without Long COVID (n = 719)	*p*
Hospitalization with ICU		117 9.5%	76 7.3%	0.05
Course assessment of the patient	VERY LIGHT-0	123 10.0%	61 8.5%	<0.001
	LIGHT-1	260 21.2%	270 37.6%	
	MEDIUM-2	412 33.6%	220 30.6%	
	SEVERE-3	432 35.2%	168 23.4%	
Fatigue		652 53.1%	1 0.1%	<0.001
Memory and concentration disturbances		221 18.0%	1 0.1%	<0.001
Anosmia and ageusia		80 6.5%	0 0%	<0.001
Hair loss		122 9.9%	1 0.1%	<0.001
Dyspnea		108 8.8%	0 0%	<0.001
Musculoskeletal pain		86 7.0%	2 0.2%	<0.001
Headache		38 3.1%	0 0.0%	<0.001
Sleep disorders, neurosis, depression, bow		33 2.7%	0 0%	<0.001

Abbreviations: ICU—intensive care unit.

**Table 4:** Lifestyle influence on Long COVID in women.

Variable		Women with Long COVID (n = 1227)	Women Without Long COVID (n = 719)	*p*
Characteristics of the study group: lifestyle				
Stimulants	Lack	1126 91.8%	662 92.0%	0.96
	Smoking	89 7.2%	50 7.0%	
	Alcohol	12 1%	7 1%	
Stress/fatigue/overwork 4 weeks before the onset of COVID-19	no	860 70.1%	506 70.4%	0.89
	yes	367 29.9%	213 29.6%	
Systematic physical and sports activity	yes	290 23.7%	148 20.6%	0.19
	no	936 76.3%	570 79.4%	

**Table 5:** Echocardiographic and MRI parameter evaluation in females with and without Long COVID.

Variable	Women with Long COVID (n = 1227)	Women Without Long COVID (n = 719)	*p*
Echocardiographic assessment			
LVEDV (mL)	97.00 (83.00–120.00)	105.00 (87.00–122.00)	0.09
LVESV (mL)	38.00 (31.00–51.00)	40.00 (30.00–48.00)	0.58
LVEF (%)	60.00 (56.00–66.00)	63.00 (57.00–68.00)	0.01
LVeSD (mm)	30.00 (25.00–32.00)	30.00 (25.00–32.00)	0.34
LVeDD (mm)	44.00 (41.00–46.00)	44.00 (41.00–46.00)	0.31
LA (mm)	37.00 (35.00–40.00)	37.00 (34.00–39.00)	0.02
Ao (mm)	30.00 (28.00–32.00)	30.00 (27.00–32.00)	0.22
IVS (mm)	10.00 (9.00–11.00)	10.00 (9.00–11.00)	0.65
A (cm/s)	10.00 (9.00–11.00)	10.00 (9.00–11.00)	0.68
RV (mm)	28.00 (26.00–30.00)	28.00 (26.00–29.00)	0.76
TAPSE (mm)	25.00 (24.00–26.00)	25.00 (24.00–26.00)	0.77
Cardiac-magnetic-resonance assessment			
LGE	76 20.4%	20 12.1%	0.02
Akinesia/hypokinesia	51 4.2%	19 2.6%	0.08

Abbreviations: LVEDV—left ventricular end-diastolic volume, LVESV—left ventricular end-systolic volume, LVEF—left ventricular ejection fraction, LVeSD—left ventricular end-systolic diameter, LVeDD—left ventricular end-diastolic dimension, LA—left atrium, Ao—aorta, IVS—interventricular septum, RV—right ventricle, TAPSE—tricuspid annular plane systolic excursion, LGE—late gadolinium enhancement, CMR—cardiac magnetic resonance.

**Table 6:** The parameters in ECG, 24 h ECG, and ABP monitoring in patients with and without Long COVID.

Variable	Women with Long COVID (n = 1227)	Women Without Long COVID (n = 719)	*p*
24 h ECG monitoring			
Heart rate (beats/minute)	75.00 (68.00–82.75)	75.00 (68.00–83.00)	0.71
PR interval	158.00 (144.00–174.50)	156.00 (141.00–172.00)	0.05
QRS	92.00 (84.00–98.00)	90.00 (84.00–98.00)	0.31
QTcB	417.00 (400.00–433.00)	414.00 (399.00–430.00)	0.009
Min day HR	57.00 (52.00–63.00)	56.00 (51.00–61.00)	0.004
Max day HR	127.00 (115.00–141.00)	127.00 (114.00–140.00)	0.76
Mean HR	74.50 (69.00–81.00)	75.00 (69.25–80.00)	0.84
ExSV	13 (1.1%)	8 (1.1%)	0.91
ExV	19 (1.5%)	6 (0.8%)	0.17
ECG assessment			
Tachycardia > 100 min	41 3.3%	20 2.8%	0.49
QRS fragmentation	153 12.5%	58 8.1%	0.002
QRS ≥ 120	48 3.9%	24 3.3%	0.51
Incorrect ST-T, appendix T	79 6.4%	44 6.1%	0.78
Arrhythmias (AF, ExSV, ExV)	36 2.9%	17 2.4%	0.45
Any abnormality HR > 100, QRS ≥ 120 ms, ST-T changes, T changes, arrhythmia, QRS fragmentation	317 25.8%	138 19.2%	<0.001
24 h ABPM			
Mean systolic blood pressure	121.00 (113.28–131.80)	123.00 (113.85–131.85)	0.98
Mean diastolic blood pressure	73.00 (67.25–78.65)	73.00 (68.00–79.00)	0.75
MAP mean daily	89.80 (84.00–95.70)	89.60 (83.00–96.50)	0.34
PP mean daily	49.45 (43.00–56.30)	48.10 (42.97–55.30)	0.07
Systolic mean daily	123.00 (113.85–131.85)	121.00 (113.28–131.80)	0.32
Diastolic mean daily	73.00 (67.25–78.65)	73.00 (68.00–79.00)	0.45
MAP mean	94.00 (87.15–100.40)	94.00 (88.00–101.00)	0.88
PP mean	49.45 (43.00–56.30)	48.10 (42.98–55.30)	0.07
Systolic blood pressure night dipping	13.00 (8.00–17.38)	13.00 (8.00–18.00)	0.51

Abbreviations: ECG—electrocardiogram, ExSV—supraventricular extrasystoles, ExV—ventricular extrasystoles, AF—atrial fibrillation, HR—heart rate, MAP—mean arterial pressure, PP—pulse pressure.

**Table 7:** Results of multivariate analysis in females with median age < 53 years old.

Parameter	OR	95% CI	*p*
Severe course of infection	1.64	1.21–2.25	0.002
BMI > 24.8 (kg/m^2^)	1.33	1.01–1.74	0.039
TG/HDL ratio > 1.89	1.64	1.18–2.28	0.003
Duration of symptoms (days) > 9	1.40	1.07–1.84	0.014

**Table 8:** Results of multivariate analysis in females with median age > 53 years old.

Parameter	OR	95% CI	*p*
Severe course of infection	1.62	1.20–2.18	0.002
Sum of symptoms > 10	1.69	1.17–2.45	0.005
TG/HDL ratio > 1.89	1.55	1.17–2.06	0.002
LVEF(%) < 67	1.78	1.18–2.69	0.006

## Data Availability

The data presented in this study are available on request from the corresponding author.

## Supplementary Materials

The following supporting information can be downloaded at: https://www.mdpi.com/article/10.3390/jcm13247829/s1, Table S1: The clinical characteristics—differences between younger females < 53 median age with and without Long COVID; Table S2: Comorbidities in younger females (median age < 53) with and without Long COVID; Table S3: The course and symptoms during COVID-19 in younger females (median age < 53) with and without Long COVID; Table S4: Lifestyle influence on Long COVID in younger women (median age < 53) with and without LC; Table S5: Echocardiographic parameters evaluation in younger females (median age < 53 years) with and without Long COVID; Table S6: The clinical characteristics—differences between older females > 53 median age with and without Long COVID; Table S7: Comorbidities in older females (median age < 53 and >53) with and without Long COVID; Table S8: The course and symptoms during COVID-19 in older females (median age > 53) with and without Long COVID; Table S9: Lifestyle influence on Long COVID in older women (median age > 53) with and without LC; Table S10: Echocardiographic parameters evaluation in older females (median age > 53 years) with and without Long COVID.
