# Supplementary material for: The Influence of Long COVID on the Cardiovascular System and Predictors of Long COVID in Females: Data from the Polish Long COVID Cardiovascular (PoLoCOV-CVD) Study
Source: J Clin Med. 2024 Dec 22;13(24):7829. doi: 10.3390/jcm13247829 (PMC11677263; doi:10.3390/jcm13247829)
Supplement: Supplementary file 1 [file jcm-13-07829-s001.zip › jcm-3323043-supplementary.pdf]

Suplementary materials - Tables S1-S10 presenting comparison between younger females with and without LC and older females with and without LC

Table S1. The clinical characteristics—differences between younger females <53 median age with and without Long COVID.

	**Women with Long COVID Age <53 (n=618)**	**Women without Long COVID Age <53 (n=376)**	p
Number of weeks after COVID-19	16.14 (9.00-27.71)	10.28 (7.14-14.71)	<**0.001**
Weight (kg)	71.00 (62.00-83.00)	68.00 (60.00-80.00)	**0.006**
Height (cm)	167.00 (163.00-170.00)	165.00 (163.00-170.00)	0.24
Body Mass Index (kg/m²)	25.39 (22.23-30.11)	24.32 (21.84-28.78)	0.01
duration of symptoms (the number of days)	10 (7-15)	10 (6-14)	<**0.001**
Sum of symptoms	8.50 (6.00-11.00)	7.00 (4.00-10.00)	**<0.001**
Sum of symptops after COVID-19	6.00 (3.00-9.00)	3.00 (2.00-6.00)	<**0.001**
No comorbidities - 1	312 (50.48%)	195 (51.86%)	0.67
Symptomatic course of COVID-19	581 (94.01%)	360 (95.74%)	0.23
Home isolation	538 (87.06%)	350 (93.08%)	**0.003**
Hospitalization with pneumonia	36 (5.83%)	13 (3.46%)	0.09
TC (mg/dl)	193.00 (170.00-215.00)	192.00 (167.00-214.00)	0.63
HDL (mg/dl)	59.00 (50.00-66.00)	61.00 (52.00-69.00)	**0.01**
LDL (mg/dl)	114.00 (92.00-134.00)	112.00 (90.00-133.00)	0.56
TG (mg/dl)	90.00 (65.00-126.00)	79.50 (62.50-113.00)	**0.003**
non HDL (mg/dl)	132.00 (110.00-157.00)	130.00 (109.00-152.00)	0.35
RC (mg/dl)	18 (13-25)	16 (12-22)	**0.007**
TG/HDL (mg/dl)	1.54 (1.04-2.41)	1.31 (0.93-2.08)	0.002
Glucose (mg/dl)	94.00 (88.00-101.00)	93.00 (87.00-100.00)	0.30
D-dimers (µg/l)	0.29 (0.20-0.39)	0.29 (0.20-0.42)	0.64
D3 level (µg/l)	27.70 (20.00-38.00)	27.80 (20.00-38.00)	0.99
Calcium (mg/dl)	9.40 (9.10-9.60)	9.34 (9.09-9.52)	0.20

Abbreviations: TC – total cholesterol, HDL - High-Density Lipoprotein Cholesterol, LDL - Low-Density Lipoprotein Cholesterol, RC - remnant cholesterol, TG – triglyceride

Table S2. Comorbidities in younger females (median age<53) with and without Long COVID.

Parameter		Age <53 (n=618)	Age <53 (n= 376)	p
HA		103 (16.66%)	50 (13.29%)	0.15
DM type 2		26 (4.20%)	14 (3.72%)	0.70
Coronary artery disease		2 (0.32%)	1 (0.27%)	1.00
Myocardial infection in the past		2 (0.32%)	1 (0.26%)	1.00
Stenocardia degree according to CCS scale	0	602 (99.67%)	338 (99.73%)	0.72
	1	2 (0.33%)	1 (0.27%)	
Cardiomyopathy		2 (0.33%)	1 (0.27%)	1.00
Heart failure, NYHA class	0	614 (99.35%)	373 (99.20%)	0.50
	1	4 (0.65%)	2 (0.53%)	
	2	0 (0%)	1 (0.27%)	
	3	0 (0.00%)	0 (0.00%)	
Hyperlipidaemia		63 (10.19%)	35 (9.31%)	0.74
Asthma		57 (9.22%)	33 (8.77%)	0.90
COPD		2 (0.32%)	1 (0.27%)	1.00
Thyroid disease		103 (16.67%)	79 (21.01%)	0.08
Hashimoto		81 (13.11%)	52 (13.83%)	0.77

Abbreviations: HA – hypertension arterialis, DM – diabetes mellitus, COPD – chronic obstructive pulmonary disease

Table S3. The course and symptoms during COVID-19 in younger females (median age<53) with and without Long COVID.

**Parameter**		**Age <53 (n=618)**	**Age <53 (n=376)**	**p**
Hospitalization with ICU		5 (0.84%)	5 (0.98%)	0.52
Course – subjective assessment of the patient	VERY LIGHT - 0	70 (11.33%)	24 (6.99%)	**<0.001**
	LIGHT - 1	142 (22.98%)	150 (39.89%)	
	MEDIUM - 2	208 (33.66%)	112 (29.79%)	
	SEVERE - 3	198 (32.04%)	77 (20.50%)	
Fatigue		298 (48.22%)	1 (0.27%)	**<0.001**
Memory and concentration disturbances		113 (18.28%)	1 (0.27%)	**<0.001**
Anosmia and ageusia		46 (7.44%)	0 (0.00%)	**<0.001**
Hair loss		54 (8.74%)	0 (0.00%)	**<0.001**
Dyspnoea		46 (7.44%)	0 (0.00%)	**<0.001**
Musculoskeletal pain		34 (5.50%)	2 (0.53%)	**<0.001**
Headache		24 (3.88%)	0 (0.00%)	**<0.001**
Sleep disorders, neurosis, depression, bow		12 (1.94%)	0 (0.00%)	**0.005**

Abbreviations: ICU – intensive care unit

Table S4. Lifestyle influence on Long COVID in younger women (median age<53) with and without LC.

Parameter		Age <53		p
		Women with Long COVID <53 (n=618)	Women without Long COVID <53 (n=376)	
Stimulants	Lack	556 (89.97%)	345 (91.76%)	0.52
	Smoking	55 (8.9%)	26 (6.92%)	
	Alcohol	7 (1.13%)	5 (1.33%)	
Stress/ fatigue/ overwork 4 weeks before the onset of COVID-19	NO	385 (62.30%)	229 (60.90%)	0.66
	YES	233 (37.70%)	147 (39.10%)	
Systematic physical and sports activity	NO	447 (72.33%)	279 (74.20%)	0.51

Table S5. Echocardiographic parameters evaluation in younger females (median age<53 years) with and without Long COVID.

**Parameter**	**Age <53 (n=618)**	**Age <53 (n=376)**	**p**
LVEDV (ml)	100.00 (85.00-122.00)	108.00 (90.00-123.00)	0.20
LVESV (ml)	39.00 (33.00-53.00)	44.00 (36.00-49.00)	0.79
LVEF (%)	60.00 (56.00-65.00)	61.00 (56.00-66.00)	0.08
LVeSD (mm)	29.00 (25.00-32.00)	29.00 (25.00-32.00)	0.78
LVeDD (mm)	44.00 (42.00-47.00)	44.00 (41.00-46.00)	0.30
LA (mm)	36.00 (34.00-39.00)	35.00 (33.00-38.00)	0.08
Ao (mm)	29.00 (27.00-31.00)	28.00 (26.00-30.00)	0.06
IVS (mm)	9.00 (8.00-10.00)	9.00 (8.00-10.00)	0.93
A (cm/s)	9.00 (8.00-10.00)	9.00 (8.00-10.00)	0.93
RV (mm)	27.00 (25.00-29.00)	27.00 (25.00-29.00)	0.89
TAPSE (mm)	25.00 (24.00-27.00)	25.00 (24.00-26.00)	0.71

Abbreviations: LVEDV - left ventricular end-diastolic volume, LVESV - Left Ventricular end Systolic Volume, LVEF - left ventricle ejection fraction, LVeSD - left ventricular end-systolic diameter, LVeDD - left ventricular end-diastolic dimension, LA – left atrium, Ao – aorta, IVS - interventricular septum, RV - right ventricle, TAPSE - Tricuspid Annular Plane Systolic Excursion

Table S6. The clinical characteristics—differences between older females >53 median age with and without Long COVID.

**Parameter**	**Women with Long COVID Age >53 (n=609)**	**Women without Long COVID Age >53 (n=343)**	**p**
Number of weeks after COVID-19	17.93 (10.29-29.03)	10.28 (7.14-15.35)	**<0.001**
Weight (kg)	74.00 (65.00-83.00)	72.00 (63.00-80.00)	0.05
Height (cm)	162.00 (158.00-165.00)	163.00 (158.00-166.00)	0.62
Body Mass Index (kg/m²)	27.73 (24.84-31.23)	27.14 (24.03-30.48)	0.05
Duration of symptoms (the number of days)	14 (7-15)	10 (6-14)	**<0.001**
Sum of symptoms	8.00 (5.00-11.00)	8.00 (4.00-10.00)	**0.007**
Sum of symptoms after COVID-19	6.00 (3.00-8.00)	4.00 (2.00-7.00)	**<0.001**
No comorbidities - 1	109 (17.89%)	61 (17.78%)	0.96
Symptomatic course of COVID-19	576 (94.58%)	328 (95.63%)	0.47
Home isolation	481 (78.98%)	289 (84.26%)	**0.04**
Hospitalization with pneumonia	81 (13.3%)	36 (10.49%)	0.206
TC (mg/dl)	201.00 (168.00-233.00)	202.00 (172.00-230.00)	0.80
HDL (mg/dl)	59.00 (50.00-67.00)	59.00 (51.00-67.00)	0.30
LDL (mg/dl)	119.00 (89.00-149.00)	122.00 (91.00-144.00)	0.63
TG (mg/dl)	111.00 (80.00-148.00)	102.00 (77.00-142.00)	0.02
non HDL (mg/dl)	140.00 (110.00-171.00)	140.00 (110.00-165.00)	0.41
RC (mg/dl)	22 (16-29)	20 (14-28)	0.14
TG/HDL (mg/dl)	1.91 (1.26-2.79)	1.69 (1.17-2.67)	0.03
Glucose (mg/dl)	99.00 (92.00-108.00)	99.00 (92.00-109.00)	0.82
D-dimers (µg/l)	0.45 (0.33-0.70)	0.43 (0.31-0.71)	0.49
D3 level (µg/l)	31.00 (24.00-39.00)	32.00 (23.00-42.00)	0.67
Calcium (mg/dl)	9.50 (9.25-9.81)	9.42 (9.20-9.69)	0.25

Abbreviations: TC – total cholesterol, HDL - High-Density Lipoprotein Cholesterol, LDL - Low-Density Lipoprotein Cholesterol, RC - remnant cholesterol, TG – triglyceride

Table S7. Comorbidities in older females (median age < 53 and >53) with and without Long COVID.

**Parameter**		**Age >53 (n=609)**	**Age >53 (n=343)**	**P**
HA		315 (51.72%)	190 (55.39%)	0.27
DM type 2		93 (15.27%)	44 (12.82%)	0.30
Coronary artery disease		61 (10.01%)	27 (7.87%)	0.27
Myocardial infection in the past		16 (2.63%)	7 (2.04%)	0.57
Stenocardia degree according to CCS scale	0	596 (99.83%)	372 (99.70%)	0.68
	1	1 (0.17%)	1 (0.30%)	
Cardiomyopathy		2 (0.33%)	1 (0.30%)	1.00
Heart failure, NYHA class	0	604 (99.18%)	341 (99.41%)	0.56
	1	2 (0.33%)	2 (0.58%)	
	2	2 (0.33%)	0 (0.00%)	
	3	1 (0.16%)	0 (0.00%)	
Hyperlipidaemia		177 (29.06%)	88 (25.66%)	0.26
Asthma		76 (12.48%)	36 (10.49%)	0.36
COPD		20 (3.28%)	11 (3.21%)	0.94
Thyroid disease		151 (24.79%)	83 (24.19%)	0.83
Hashimoto		109 (17.89%)	48 (13.99%)	0.11

Abbreviations: HA – hypertension arterialis, DM – diabetes mellitus, COPD – chronic obstructive pulmonary disease

Table S8. The course and symptoms during COVID-19 in older females (median age>53) with and without Long COVID.

		**Age >53 (n=609)**	**Age >53 (n=343)**	**p**
Hospitalization with ICU		4 (0.68%)	1 (0.29%)	0.65
Course – subjective assessment of the patient	VERY LIGHT - 0	53 (8.70%)	37 (9.84%)	**<0.001**
	LIGHT - 1	118 (19.38%)	120 (34.99%)	
	MEDIUM - 2	204 (33.50%)	108 (31.49%)	
	SEVERE-3	234 (38.42%)	91 (26.53%)	
Fatigue		354 (58.13%)	0 (0.00%)	**<0.001**
Memory and concentration disturbances		108 (17.73%)	0 (0.00%)	**<0.001**
Anosmia and ageusia		34 (5.58%)	0 (0.00%)	**<0.001**
Hair loss		68 (11.17%)	1 (0.29%)	**<0.001**
Dyspnoea		62 (10.18%)	0 (0.00%)	**<0.001**
Musculoskeletal pain		52 (8.54%)	0 (0.00%)	**<0.001**
Headache		14 (2.30%)	0 (0.00%)	**<0.001**
Sleep disorders, neurosis, depression, bow		21 (3.45%)	0 (0.00%)	**<0.001**

Abbreviations: ICU – intensive care unit

Table S9. Lifestyle influence on Long COVID in older women (median age>53) with and without LC.

Parameter		**Women with Long COVID (n=609) >53**	**Women without Long COVID (n=343) >53**	**P**
Stimulants	Lack	570 (93.60%)	317 (92.42%)	0.63
	Smoking	34 (5.58%)	24 (6.99%)	
	Alcohol	5 (0.82%)	2 (0.58%)	
Stress/ fatigue/ overwork 4 weeks before the onset of COVID-19	NO	475 (78.00%)	277 (80.75%)	0.31
	YES	134 (22.00%)	66 (19.25%)	
Systematic physical and sports activity	NO	490 (80.30%)	291 (84.84%)	0.13

Table S10. Echocardiographic parameters evaluation in older females (median age>53 years) with and without Long COVID.

**Parameter**	**Age >53 (n=609)**	**Age >53 (n=343)**	**P**
LVEDV (ml)	90.00 (80.00-109.00)	96.00 (80.00-109.00)	0.33
LVESV (ml)	33.00 (24.00-44.00)	34.00 (27.00-46.00)	0.66
LVEF (%)	61.00 (57.00-67.00)	63.00 (59.00-69.00)	0.02
LVeSD (mm)	30.00 (25.00-32.00)	30.00 (26.00-32.00)	0.57
LVeDD (mm)	44.00 (41.00-46.00)	43.00 (41.00-47.00)	0.65
LA (mm)	38.00 (36.00-42.00)	38.00 (36.00-41.00)	0.22
Ao (mm)	30.00 (28.00-33.00)	30.00 (29.00-33.00)	0.68
IVS (mm)	10.00 (9.00-11.00)	11.00 (9.00-11.00)	0.51
A (cm/s)	10.00 (9.00-11.00)	11.00 (9.00-11.00)	0.53
RV (mm)	28.00 (26.00-30.00)	28.00 (26.00-30.00)	0.67
TAPSE (mm)	25.00 (23.00-26.00)	24.00 (23.00-25.00)	0.81

LVEF - left ventricle ejection fraction, LVeSD - left ventricular end-systolic diameter, LVeDD - left ventricular end-diastolic dimension, LA – left atrium, Ao – aorta, IVS - interventricular septum, RV - right ventricle, TAPSE - Tricuspid Annular Plane Systolic Excursion
